# Utilizing Stereotactic Spine Navigation for Posterior Partial Vertebrectomy in an En Bloc Resection of a Superior Pulmonary Sulcus Tumor Invading the Thoracic Vertebrae: A Technical Note

**DOI:** 10.7759/cureus.3303

**Published:** 2018-09-14

**Authors:** Mateo Ziu, Jeffrey I Traylor, Jason Paxman, Allison Gorrebeeck, Daniel L Fortes

**Affiliations:** 1 Department of Surgery & Perioperative Care, The University of Texas at Austin, Dell Medical School, Austin, USA; 2 Medical Student, The University of Texas at Austin, Dell Medical School, Austin, USA; 3 Department of Internal Medicine, Dell Seton Medical Center at The University of Texas, Austin, USA; 4 Department of Surgery and Perioperative Care, The University of Texas at Austin, Austin, USA

**Keywords:** en bloc resection, non-small cell lung cancer, superior sulcus tumor, stereotactic spine navigation, spine tumor, o-arm

## Abstract

Prior to the development of en bloc techniques, vertebral invasion by non-small cell lung cancer (NSCLC) had been considered a relative contraindication to surgical intervention. However, reports in the literature have demonstrated increased progression-free survival with the use of neoadjuvant chemotherapy followed by anterior en bloc resection of the residual tumor. Stereotactic spine navigation has been shown to improve accuracy during complex vertebral osteotomies, improving patient outcomes. We report a 53-year-old woman with an NSCLC in the left upper lobe, a periosteum attachment of the second and third thoracic vertebrae (T2 and T3, respectively), and an infiltration of the corresponding nerve roots. We describe a surgical approach for the resection of NSCLC with vertebral infiltration utilizing stereotactic spine navigation and intraoperative computed tomography (CT) (O-Arm, Medtronic, Minneapolis, Minnesota, US) for a posterior approach laminectomy, osteotomy, and partial vertebrectomy, followed by trans-thoracic en bloc resection of a superior pulmonary sulcus tumor with nerve root infiltration. Posterior approach vertebral osteotomy and en bloc resection for superior sulcus NSCLC infiltrating the vertebrae utilizing stereotactic spine navigation and intraoperative CT (O-Arm) is a viable alternative to the traditional anterior approach.

## Introduction

Non-small cell lung cancer (NSCLC) comprises the majority of lung cancer diagnosed in the United States and confers an overall five-year survival of less than 18% [1]. For patients with NSCLC, surgical resection is considered first-line therapy in stages I, II, and N0 or N1 stage IIIa disease. Resectability is determined by the degree of tumor invasion into the vertebral body, which historically has been considered a relative contraindication to surgery due to poor long-term survival [2]. However, data has shown improved progression-free survival with neoadjuvant chemotherapy followed by an anterior en bloc approach [3-5]. Here, we describe the use of stereotactic spine navigation and intraoperative computed tomography (CT) (O-Arm, Medtronic Corporation, Minneapolis, Minnesota, US) for guidance of a complex posterior sagittal vertebral osteotomy in order to perform a partial vertebrectomy of the second and third thoracic vertebrae (T2 and T3, respectively), followed by en bloc resection of a superior sulcus NSCLC with nerve root infiltration.

## Case presentation

**Figure 1:**
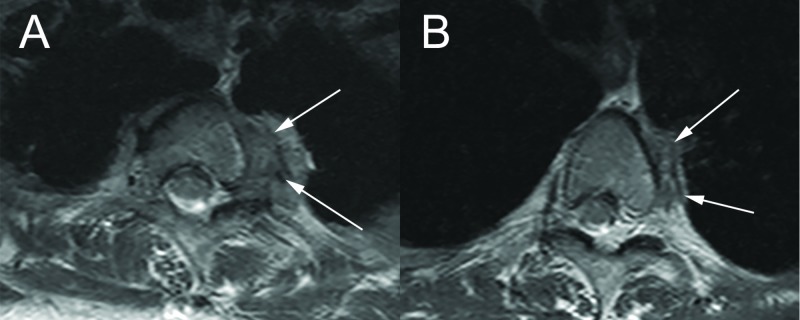
Axial MRI of T2 and T3 Thoracic Vertebrae Axial T2W magnetic resonance (MR) images of the thoracic spine. On the left of the vertebral bodies (A: T2 vertebral body. B: T3 vertebral body), the tumor mass is delineated by white arrows. MRI: magnetic resonance imaging

Our patient is a 53-year-old woman with a posterior, stage IIB NSCLC in the left upper lobe who received neoadjuvant carboplatin, pemetrexed, and radiotherapy. One year following diagnosis, the patient was found to have tumor attachment to the T2 and T3 vertebrae (Figures 1A-1B ) and infiltration of the corresponding nerve roots following complaints of severe left back pain and left axillary numbness and paresthesia. As a result, the neurosurgery and thoracic surgery services were consulted to discuss management. Based on the magnetic resonance imaging (MRI) studies, there was no direct tumor invasion of the left brachial plexus or subclavian vessels. After discussing different treatment options, a posterior approach, image-guided T2 and T3 osteotomy followed by transthoracicen blocresection of the thoracic tumor was recommended. The patient agreed to the operation and informed consent was obtained.

**Figure 2:**
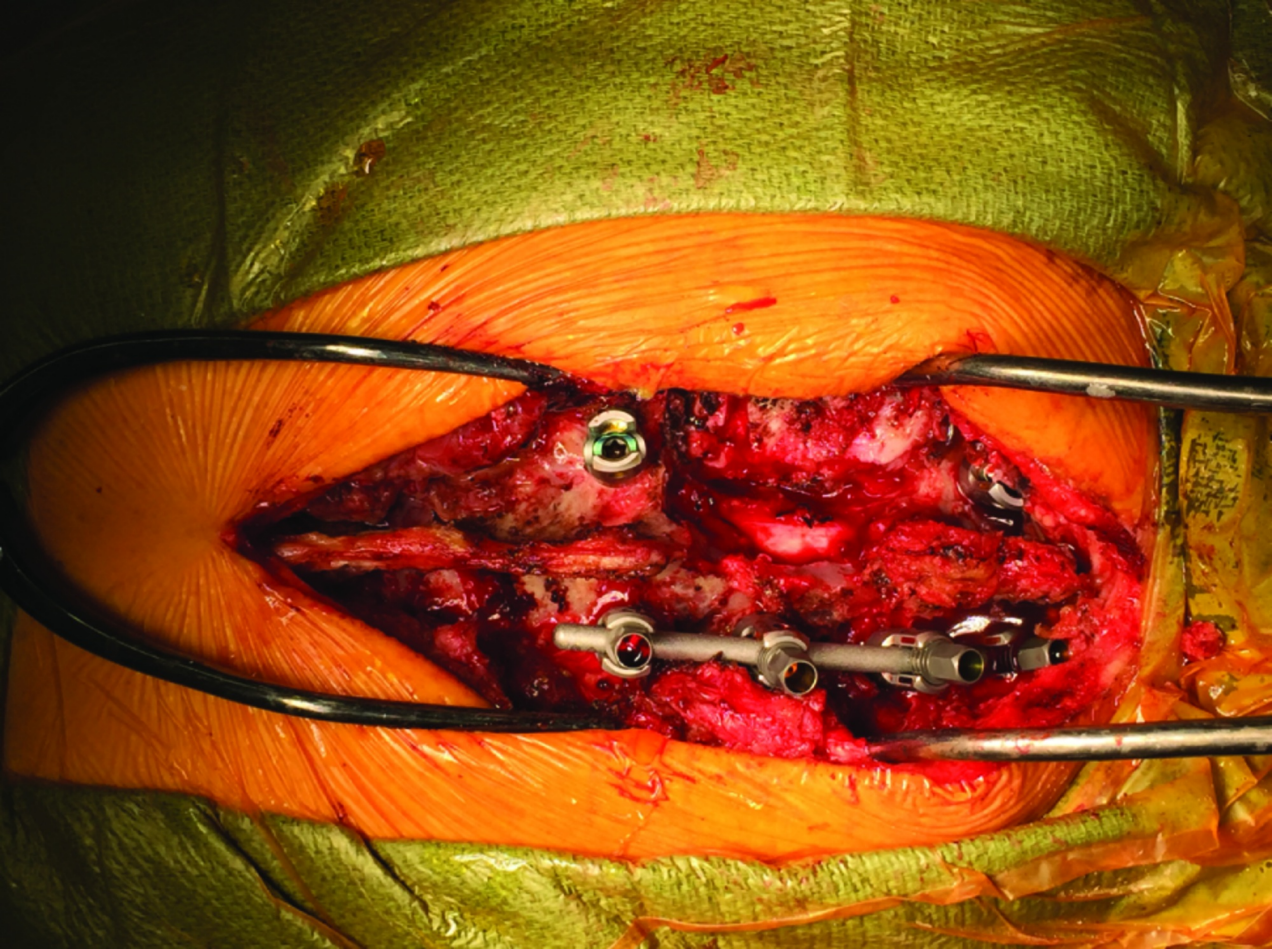
T1-T4 Vertebrae Instrumentation Instrumentation placement, posterior view, following osteotomy of T2-T3.

**Figure 3:**
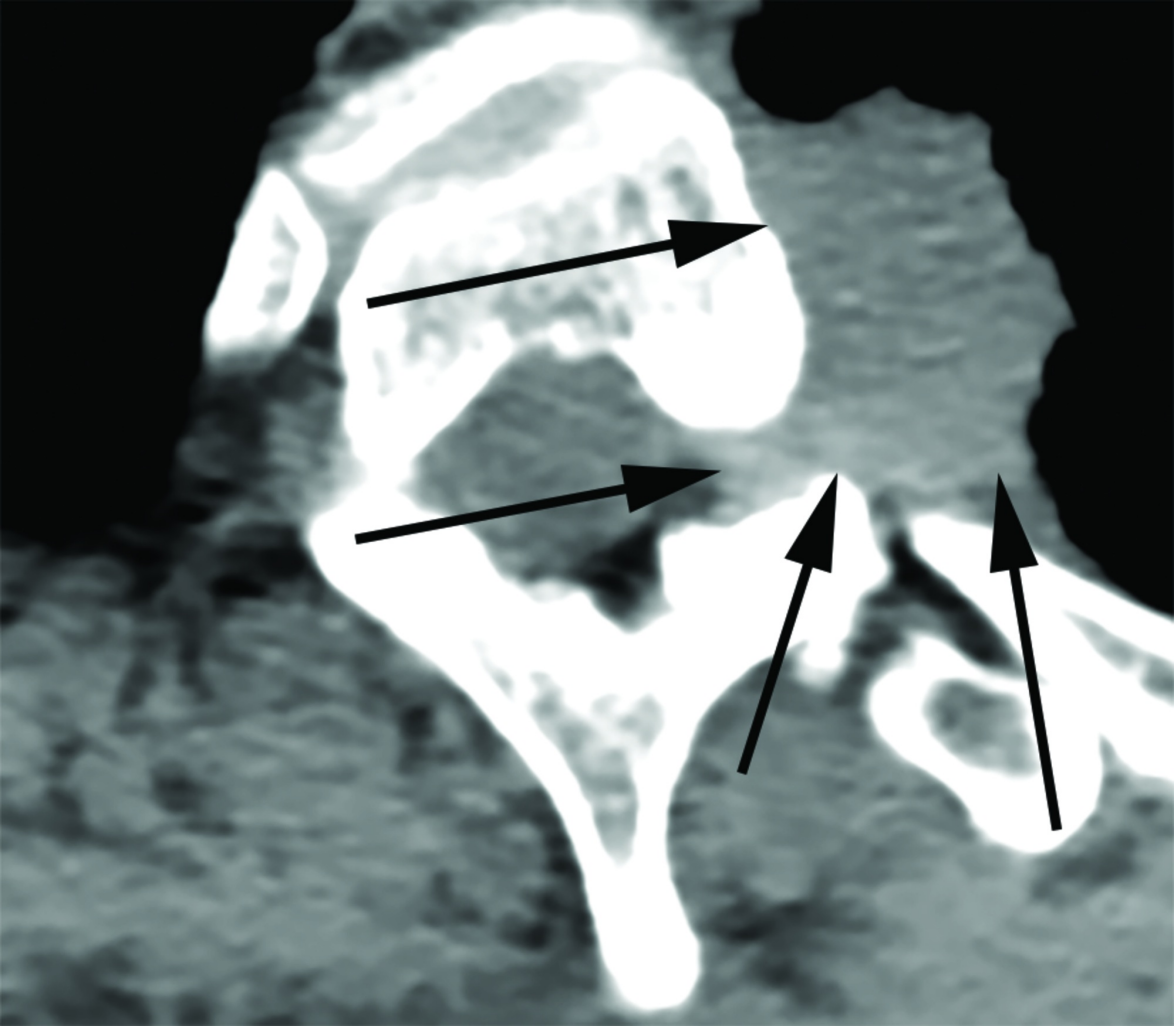
Intraoperative Axial CT of the T2 Vertebra Axial computed tomography (CT) image taken of the T2 vertebra with the O-Arm for anticipated osteotomy. Arrows delineate the tumor mass. O-Arm: Medtronic Corporation, Minneapolis, Minnesota, US

**Figure 4:**
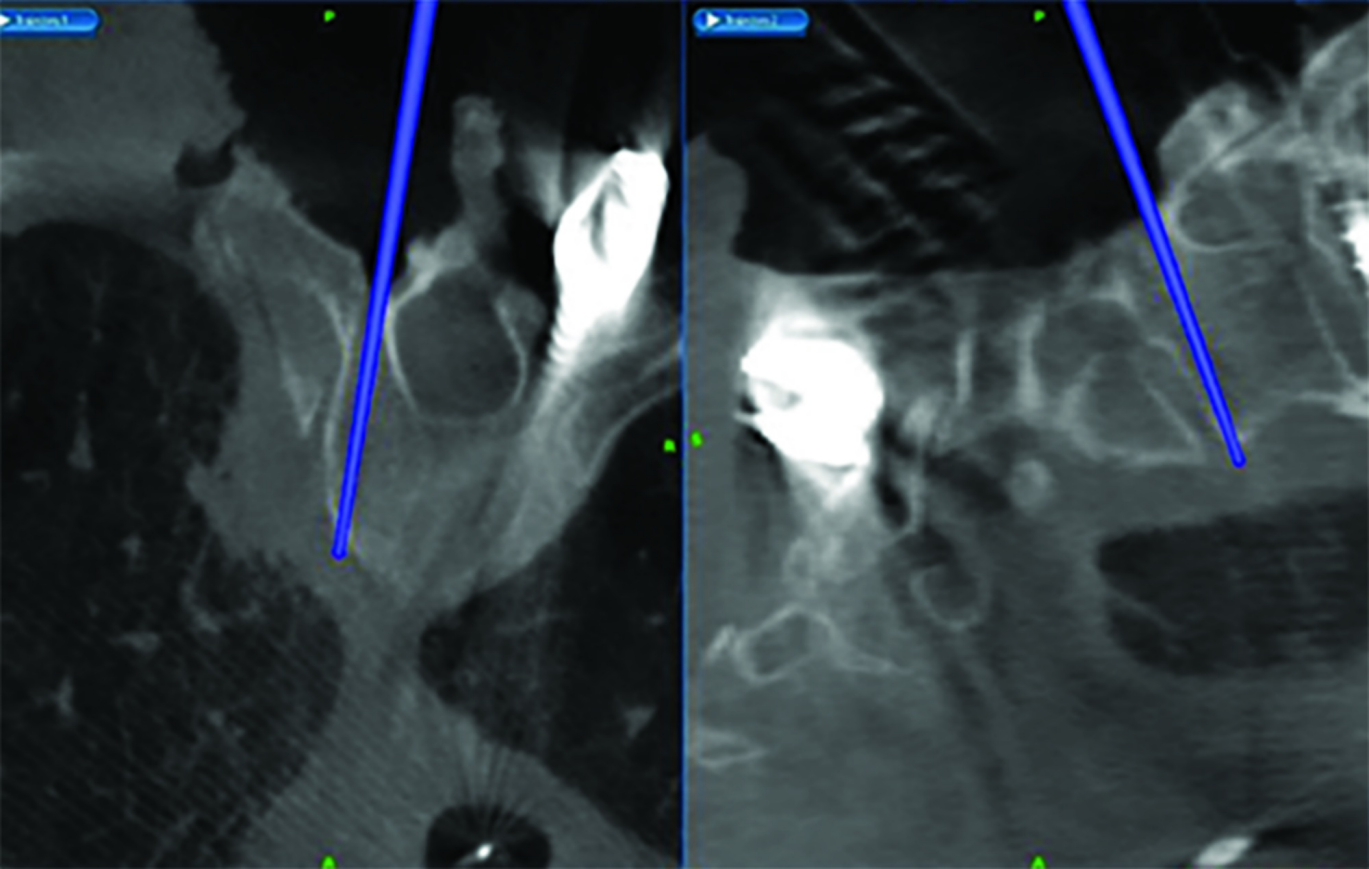
Spine Navigation Probe Spine navigation-station screen shot depicting position of navigation probe on simultaneous axial (left) and sagittal (right) planes following osteotomy.

**Figure 5:**
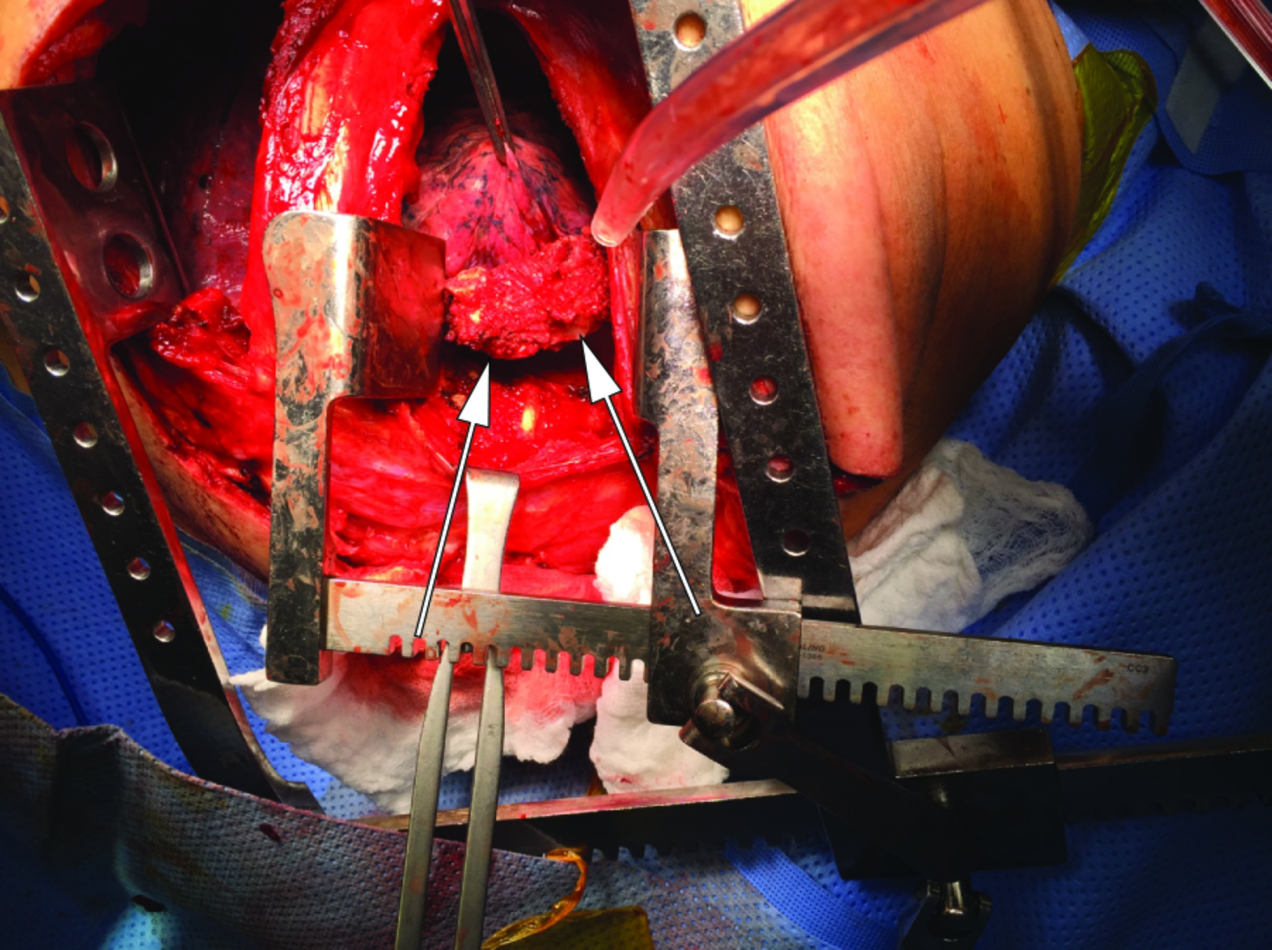
Resected En Bloc Tumor with T2 and T3 Vertebrae Resected vertebral body is shown attached to the primary tumor via thoracotomy. Arrows show the vertebral bodies with tumor attached.

**Figure 6:**
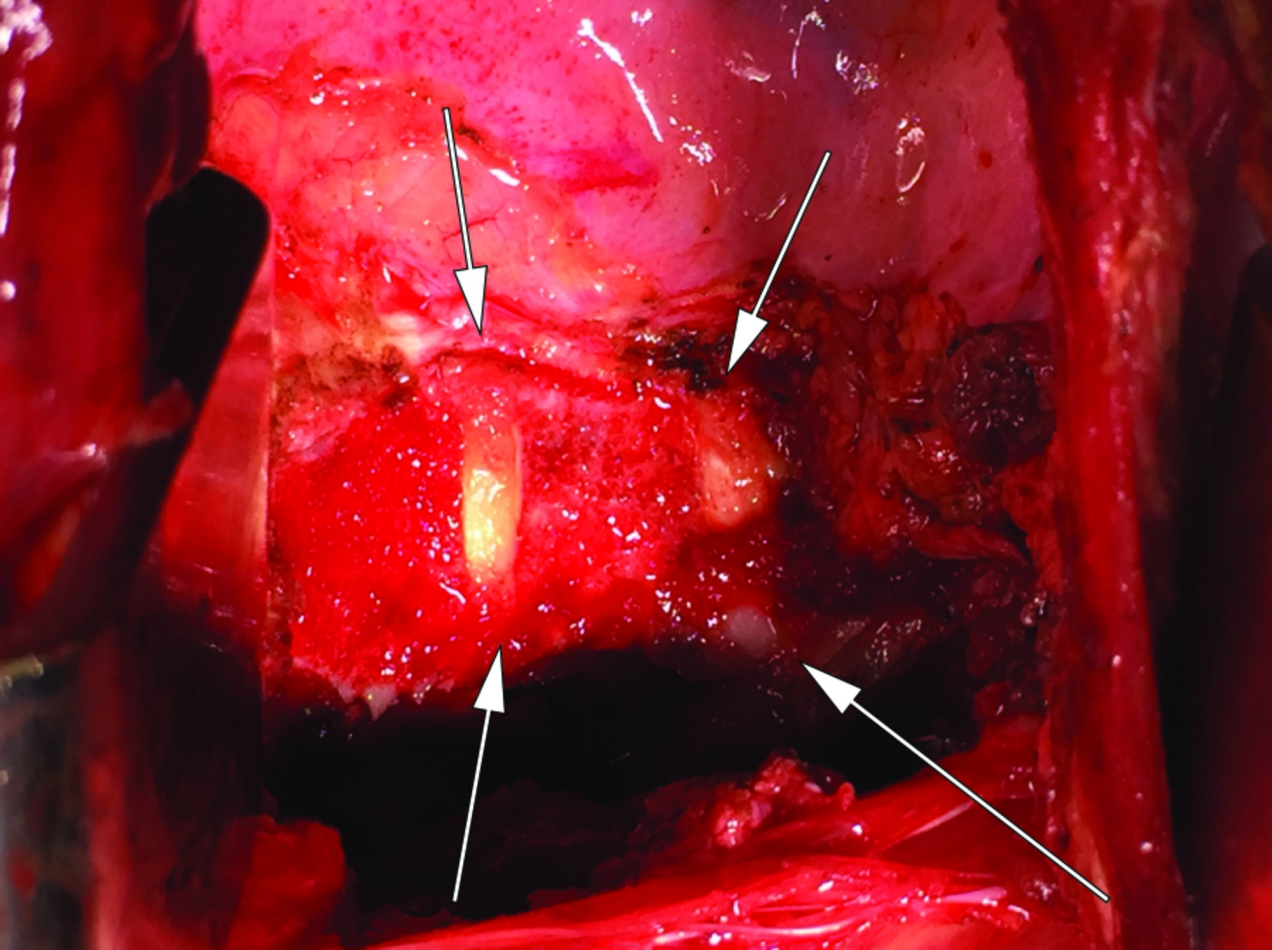
Resection Cavity A closer view of the resection cavity following *en bloc *removal via thoracotomy incision. Arrows delineate the margins of resection.

**Figure 7:**
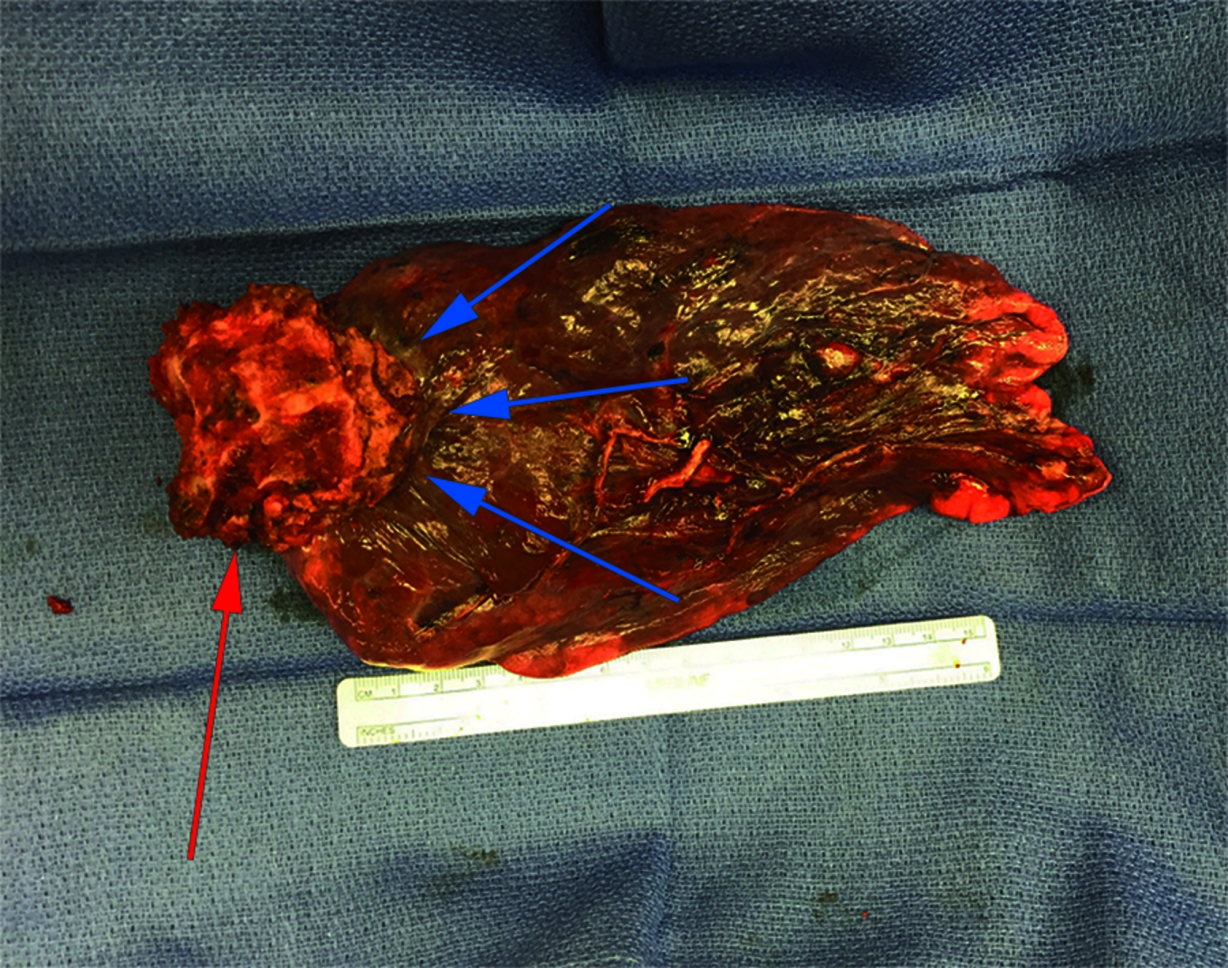
En Bloc Tumor Specimen Anterior view of the tumor with attached portion of the T2-T3 vertebral bodies following *en bloc *resection. The red arrow points to the T2 vertebra while the blue arrows delineate the tumor mass.

After the initial incision was made in the operating room, we exposed the spinal processes, lamina, and transverse processes from T1 through T5 and 5 cm of the ribs from the left costovertebral junction on the side of the tumor. At this point, images were taken with the O-Arm(Medtronic Corporation, Minneapolis, Minnesota, US). We then registered the patient's spine to the StealthStation (Medtronic Corporation, Minneapolis, Minnesota, US) navigation suite using the stereotactic probe. Then, using spine navigation, the instrumentation was placed at the level above and below the tumor-infiltrated vertebrae. We did not place screws on the left T2 and T3 vertebrae (Figure 2). The spinous processes and lamina of T2 and T3 were removed and the nerve roots exposed on the left. We noticed an infiltration of the ganglia by the primary tumor that was in continuity with the ribs on the left side. We then proceeded with resection; the T2 and T3 nerve roots were tied with a 2-0 silk tie and cut 2 mm from the tie on the extradural portion of the root. On the spine-navigation station, we determined the plane of osteotomy of the vertebral bodies of T2 and T3 (Figure 3). We anticipated disconnecting the portion of the vertebral body infiltrated by the tumor from the rest of the vertebral body, leaving it in situ for removal with the entire lung tumor in the second stage of the procedure. During the planning of our trajectory, we considered that the periosteum of the vertebrae had been infiltrated. We wanted to resect starting from the point where the tumor had infiltrated the nerve root projecting then on the anterior portion of the vertebra, planning a resection margin free of tumor from the infiltrated periosteum. Using an ultrasonic Sonopet with a serrated knife bone cutter tip (Stryker Corporation, Kalamazoo, Michigan, US), the osteotomy of the vertebral bodies of T2 and T3 was performed following the planned trajectory with the intent to leave the mass intact and avoid entering the tumor capsule. As the osteotomy was performed, we intermittently used the navigation probe to confirm our trajectory. We continued cutting through the bone with the Sonopet blade until soft tissue was felt underneath. The location was then confirmed with stereotactic spine navigation images (Figure 4). Here, we proceeded with cutting the second and third ribs 5 cm from the midline. After we confirmed that the ribs, together with the portion of the vertebral body wall infiltrated with tumor and the nerve root, were completely freed posteriorly, we proceeded with standard arthrodesis. We separated the muscle from the fascia to mobilize the tissue and closed the tissue with layered sutures. Following closure, the en bloc resection of the left upper lobe of the lung with the lateral portions of the T2 and T3 vertebral bodies and corresponding ribs together with complete mediastinal lymphadenectomy was performed through a posterolateral standard thoracotomy incision (Figures 5-7).

The final surgical pathology report revealed moderately differentiated, acinar predominant stage IIB adenocarcinoma with a single focus, all three resected lymph nodes, and six margins were negative for tumor. The nearest margin was 4 mm from the tumor, confirming the accuracy of our planned trajectory on the StealthStation. One year later, the patient was doing well, with no evidence of tumor recurrence. The patient declined any further therapy during that time and was subsequently lost to follow-up.

## Discussion

Tumors in the superior pulmonary sulcus have a particular propensity to invade the spine, historically considered to be a contraindication to surgical resection [2]. However, in 1996, Grunenwald et al. demonstrated the utility of an en bloc approach for the resection of these tumors and later showed an associated improvement in long-term survival [4,6]. These positive results were further echoed by numerous reports in the following years [7-13]. In 2004, Kent et al. described a single-staged posterior vertebrectomy followed by anterior en bloc resection of a superior sulcus tumor [14]. Later, Smitherman et al. applied frameless stereotactic navigation to complete a multi-level sagittal vertebral osteotomy for a giant cell tumor, claiming that real-time three-dimensional imaging made the procedure possible by avoiding adjacent critical vascular structures [15]. Furthermore, Mody et al. contributed to the sequence of en bloc resection by splitting up the procedure into two stages, allowing for completion in a standard day [16]. Kobayashi et al., who published their case report just a few days after we performed our case, applied the use of an O-Arm stereotactic navigation system to partial vertebrectomy for lung cancer adjacent to the thoracic spine, stating the O-Arm permitted accurate confirmation of the surgical margins of the osteotomy via a posterior approach [17].

In our case, the lack of direct tumor invasion into extraforaminal, proximal neurovascular structures coupled with the precision provided by stereotactic spine navigation allowed for posterior vertebral osteotomy to facilitate an anterior en bloc resection. Specifically, the navigation probe allowed for accurate prediction of the trajectory of the ultrasonic aspirator, along with real-time measurement of depth throughout the drilling process. Though this approach is similar to the process described by Kent et al., we utilized stereotactic spine navigation for additional precision to avoid entering the periosteum of the vertebrae and tumor capsule [14]. A resection of the thoracic wall was deemed unnecessary due to the lack of involvement by the tumor from preoperative imaging. A posterior osteotomy followed by a posterolateral thoracotomy was thus deemed sufficient for en bloc tumor removal with the medial portion of the ribs, the infiltrated nerve root, and the portion of the vertebrae. Other techniques in the literature for the resection of tumors invading the spine have either followed Grunenwald’s anterior approach or a posterior approach for tumors not invading the vertebral bodies [4]. Kobayashi et al. are the first to describe the utilization of intraoperative O-Arm stereotactic navigation for performing a posterior osteotomy of the vertebral body, freeing the posterior margin of the tumor to allow for subsequent en bloc removal from a lateral approach [17]. While Kobayashi’s team utilized an osteotome, we utilized an ultrasound aspirator drill that led to a minimal blood loss, precise cutting margins, and reduced torque on the vertebral body. Furthermore, we believe that the use of the ultrasonic aspirator reduces the number of bony particles dispersed into the surgical cavity as often occurs when standard high-speed drills are used.

With this case, we contribute to the growing body of literature a description of the utility of intraoperative stereotactic navigation for en bloc resection of extra-spinal tumors invading the vertebral column. This procedure can be performed in one day or staged on two different days depending on the logistics of the healthcare facility and surgical team workflow. It is our hope that stereotactic navigation will be viewed as a powerful tool for the relief of tumor burden from complicated anatomical structures in a safe and reliable manner.

## Conclusions

The use of stereotactic spine navigation enabled a posterior approach vertebral osteotomy followed by a lateral approach for the resection of a superior pulmonary sulcus NSCLC, allowing for precise removal despite the proximity of neurovascular structures. This technique potentially simplifies the en bloc resection of thoracic tumors infiltrating vertebral bodies.
